# Complete genome sequence of the *Streptococcus parauberis* lytic bacteriophage JYU001

**DOI:** 10.1128/mra.00036-26

**Published:** 2026-05-12

**Authors:** Young-Ung Heo, Ju-Yeop Lee, Hyo-Young Kang, Jiyeon Park, Thanh Luan Nguyen, Do-Hyung Kim

**Affiliations:** 1 Department of Aquatic Life Medicine, College of Fisheries Sciences, Pukyong National University 34998, Busan, South Korea; 2 Department of Science, Technology and International Affairs, HUTECH University 638418, Ho Chi Minh City, Vietnam; Portland State University 6685, Portland, Oregon, USA

**Keywords:** bacteriophages, bacteriophage therapy, *Streptococcus parauberis*

## Abstract

We report the complete genome sequence of *Streptococcus parauberis* bacteriophage JYU001 (*Caudoviricetes*), a lytic phage with a 36,295 bp dsDNA genome (35.27% G+C) encoding 69 predicted CDSs. No tRNA genes, integrase genes, antimicrobial resistance genes, or virulence factors were detected. A lytic lifestyle was also predicted by PhaTYP.

## ANNOUNCEMENT

*Streptococcus parauberis* bacteriophage JYU001 was isolated in 2022 from aquaculture seawater collected at an olive flounder farm in Jeju, Republic of Korea (33.3252075 N, 126.8401822 E), where *S. parauberis* is a recurrent pathogen often associated with antibiotic treatment challenges. The seawater sample was filtered (0.2 μm), enriched in brain heart infusion broth (Oxoid, UK) with *S. parauberis* serotype I strain SPOF19J5 at 28°C for 48 h, centrifuged, and filtered (0.22 μm), from which the phage was obtained using the double-layer agar method at 28°C for 24 h. Individual plaques were purified through three rounds of plating. A plaque-purified phage suspension was then propagated in the host culture under shaking conditions for 24 h. The culture was clarified by centrifugation and membrane filtration to remove bacterial debris, yielding a high-titer lysate of approximately 10⁹ PFU/mL.

Phage DNA was extracted from the lysate using a phenol-chloroform protocol (1) and quantified with a Qubit 3.0 fluorometer (dsDNA HS kit). A sequencing library was prepared with the TruSeq Nano DNA kit (Illumina, USA) and sequenced on the Illumina NovaSeq 6000 platform (151 bp paired-end mode). While approximately 16 million read pairs were initially generated, 500,000 read pairs were randomly subsampled using Seqtk v1.5 (2) to improve computational efficiency and minimize potential assembly errors associated with excessive sequencing depth. Read quality was inspected with FastQC v0.12.1 (3), and trimming was performed using Trimmomatic v0.38 (4). *De novo* assembly was generated with SPAdes v4.2.0 (5) and evaluated using QUAST v5.2.0 (6), which indicated a single contig with no ambiguous bases (0 Ns) and no detected misassemblies. Genome termini were predicted using PhageTerm v1.0.12 (7) implemented in Galaxy, indicating a COS (3′) packaging mechanism with defined cohesive ends. Genome annotation was performed using Pharokka v1.5.3 (8), which incorporates PHANOTATE for gene prediction and PHROGs database for functional annotation, and annotations were refined with HHpred (PDB mmCIF70, Pfam-A, and NCBI CDD database) (9). The presence of tRNA genes, antimicrobial resistance genes, and virulence factors was assessed using tRNAscan-SE (10), CARD (11), and VFDB (12). Phage lifestyle was evaluated using PhaTYP (13). All tools were run with default parameters unless otherwise specified.

The complete genome of JYU001 comprises a 36,295 bp linear double-stranded DNA molecule with a G+C content of 35.27%. *De novo* assembly generated a single contig (N50, 36,295 bp) with an average sequencing depth of approximately 4,160×. A total of 69 coding sequences were identified, including genes associated with structural components, packaging, nucleic acid metabolism, lysis, tail assembly, and transcriptional regulation. Thirty-nine predicted proteins were annotated as hypothetical. No integrase genes were detected, and PhaTYP also predicted a lytic lifestyle. In addition, no tRNA genes, antimicrobial resistance genes, or virulence factors were identified.

JYU001 represents a genomic resource for understanding *S. parauberis* phage biology and may contribute to future phage-based approaches for managing streptococcal infections in aquaculture.

## Data Availability

The complete genome sequence of *Streptococcus* bacteriophage JYU001 has been deposited in GenBank under accession number PX759544. The raw sequencing reads have been deposited in the NCBI Sequence Read Archive (SRA) under BioProject PRJNA1395703 and BioSample SAMN54370109, with SRA run accession number SRR36648891.
